# Extra Supplement.—The Nursing Mirror

**Published:** 1894-03-17

**Authors:** 


"The Hospital" Nursing Mirror.

Being the Extra Nursing Supplement of "The Hospital" Newspaper.

[Contributions for this Supplement should be addressed to the Editor, The Hospital, 428, Strand, London, W.C., and should have the word "Nursing" plainly written in left-hand top corner of the envelope.]

### News from the Nursing World.

### SUBJECTS FOR SERMONS.

A modest suggestion has been made by the Church Sanitary Association to the effect that on one Sunday in the year the cause of hygiene and sanitation should be advocated from the pulpit. Perhaps in instituting a Sanitation Sunday it may be well at first to ask only for one in the year, but an association aspiring to give due prominence to its objects must be ready to put these forward daily rather than annually. Cleanliness, light, air, pure water, and proper food are surely essential for securing those healthy bodies in which sound minds and morals are likely to find the best home.

### BOLD THROUGH IGNORANCE.

"Just a drop to keep 'im quiet can't do no harm!" says a certain poor woman, one of a class familiar to the district nurse in large towns, as she gives her puny baby a sip of the cordial which she finds "comforting" to her own ill-nourished body. When nurse remonstrates, she not infrequently finds her arguments as unintelligible to her hearer as if she spoke a foreign language. The colliers' wives, in North Wales, appear to give morning doses of laudanum to their infants, which suffice to keep the unfortunate little creatures unconscious until their own return from work in the evening. Surely an antidote for this horrible system of drugging would be found in proper well-cooked food, and in some knowledge of the common laws of health. Until the poor mothers are better instructed as to the dangers to which they expose themselves and their children, they will see no reason for abandoning a practice which ensures to themselves the maximum of peace at the minimum expenditure of trouble.

### DIGNITY IN DANGER.

"Was not it just perfect to go steaming away across the ocean? I can't imagine anything grander!" gushingly remarked a girl who had always lived inland. "Oh! it's nice enough," replied the nurse, just come off a long voyage, "but as for the grandeur! well, that's apt to be lost sight of when your companion happens to be taken ill in a top berth and has to be visited by a dignified medical officer who climbs up a ladder to feel her pulse whilst he tries to maintain his professional demeanour as well as his equilibrium!"

### TRAINED NURSING IN WORKHOUSES.

The Northern Workhouse Nursing Association, which deals with seventeen counties, held its annual meeting at Carlisle on the 7th inst. under the presidency of the Bishop. It was reported that only forty out of 250 unions have seen their way to subscribe, although the Local Government Board makes no objection to the guardians giving such subscriptions out of the public funds. It is to be hoped that the value of the trained nurses supplied by this admirable association will soon rouse a more liberal spirit in those appointed to guard the interests of the sick poor.

### GOOD WORK AT ALFRETON.

The work done by the Queen's nurse, Miss Rowley, at Alfreton, is particularly satisfactory from having been bravely undertaken and successfully carried out in the face of both difficulty and opposition. The vicar, Mr. Hervey, set the movement on foot, and is certainly to be congratulated on his determination to secure a fully-trained nurse. The committee, also realising the need, have worked well and energetically; and in a neighbourhood where accidents frequently happen among the colliers, the value of a nurse's services can hardly be over-estimated.

### "IN THE NURSE'S ABSENCE."

A tragic occurrence has recently brought to the knowledge of the public an extraordinary case of under-nursing at the Birmingham Small-pox Hospital. On night duty one ward containing twenty-one and another seventeen patients were under the charge of one nurse, when a poor girl only twenty-four years of age became so violently delirious that, for the sake of the other patients, the medical officer ordered her removal to a special cottage, giving directions that she was not to be left. A sleeping draught was administered, but some three hours later the unfortunate girl became again restless. The nurse still retained charge of the other two wards, and the night superintendent, Sister Bradbury, put a straight waistcoat on the delirious girl. In spite of this, during the temporary absence of the nurse, the patient managed to escape, committing suicide by drowning herself in the canal. No wonder the Coroner's jury found "that adequate precautions were not taken," and "do not consider the blame should rest on Nurse Lacey alone." The nursing staff at isolation hospitals should certainly provide adequate attendance and intelligent treatment for delirious patients who are temporarily irresponsible for their actions. Solitude and a straight waistcoat are but poor substitutes for skilled nursing.

### THE VISITING NURSE.

Under the somewhat indefinite title of the "Practitioner Daily Nurse," the Coventry Nursing Institution is now prepared to supply a visiting nurse to those patients who require the occasional services of a fully-trained woman, but yet do not need her full time. She should doubtless be to the class who can afford to pay for skilled attendance, exactly what a qualified Queen's nurse is to the poor. The plan of the daily nurse has long been in operation in London, and probably in other large towns, and without doubt it possesses distinct advantages, besides being economical for individual employers. On the other hand, it is very difficult for a visiting nurse to meet the wishes of three or four paying patients, who seem inclined to fancy that "nurse ought to come to each of them first," and that she certainly ought not to "hurry away" to another patient whose own particular doctor also urgently requires her presence.

### DISTRICT NURSES FOR NOTTINGHAM.

A largely attended public meeting was recently held in Nottingham to consider the question of providing trained nurses for the sick poor. It is pleasant to find that the speakers, who included the Bishop of Southwell, Lord Belper, Canon Trebeck, and Dr. William Ransom, agreed unanimously as to the necessity for securing properly trained nurses. Affiliation with the Queen's Jubilee Institute would be a step well worthy of consideration by the influential committee appointed to go into the plans and to decide upon the form which the new organisation should take.

### WANTED—A COTTAGE HOSPITAL.

There seems no doubt that the Cottage Hospital, which has again been under discussion at Barry, would become an immense boon to the neighbourhood, but so far no practical suggestions for securing one appear to have been made. The work of the Barry and District Nursing Association continues to be thoroughly appreciated, and we hope it will not be long ere suitable hospital accommodotion for urgent cases of accident or illness will be attainable without need for the sufferers journeying so far as Cardiff. The victims of serious dock casualties must assuredly find the transit most trying, however efficiently it be managed.

### A PROSPEROUS SOCIETY.

A most gratifying financial report was presented at the annual meeting of the Derry District Nursing Society, which proves that although the year has been commercially a bad one, those interested in the care of the sick poor have not lost sight of their claims. Each report is more satisfactory than the last, and there is every prospect of the useful work of the association (which is affiliated with the Queen's Jubilee Institute) continuing to grow and to prosper each year.

### GAIETY AT GLASGOW.

Lord Provost and Mrs. Bell gave a conversazione and dance to the nurses of Glasgow, at the City Chambers, on the 5th inst., the occasion being the celebration of the centenary of the foundation of the Royal Infirmary. The Lord Provost and Dr. Donald Macleod in their excellent speeches alluded with much spirit to the inadequate support which the hospitals received from the rich inhabitants of Glasgow. He charitably attributed the failure in this respect rather to want of thought than want of heart.

### MIXED MOTIVES.

Cigarette-smoking appears to be attended with considerable peril in San Francisco to young people of both sexes who patronise the habit. Analysis proves the presence of ammonium chloride and many other ingredients besides tobacco in the cigarettes purchased. The Board of Education has been considering the subject, and clergy and others are interesting themselves in a crusade against the habit of immoderate smoking indulged in by the precocious youth of San Francisco. It is proposed to discourage all smoking by persons under twenty-one years of age, which is certainly a reasonable suggestion in the eyes of parents and schoolmasters, as well as of doctors. Any prohibitive measures will, however, assuredly fail to secure sympathy from the advanced young women of the period, who, in all civilised countries, assume that the habit of smoking proves their superiority over their more timid sisters. Scorning the accusation that the custom merely establishes them on the level of the young men they profess to despise, they assert that they value tobacco for its soothing properties. If that were its sole attraction it would surely, like sleeping draughts or hot negus, be "taken at bedtime," instead of ostentatiously paraded in the full glare of daylight by emancipated damsels!

### PLEASANT ENVIRONS.

The difficulties and the pleasures of the Nursing Sisters' lives in India are often freely discussed, but their actual surroundings are seldom described. Yet the latter are often well worth talking about, for our sisters on foreign service are able to make their bungalows cosy and pretty. When two or three ladies are quartered together, and reside a little way apart from the hospital, though within easy reach of it, they may be frequently congratulated upon their pleasant-looking home as well as on the charming garden surrounding it.

### AUSTRALIAN HEALTH SOCIETY.

The Australian Health Society, according to its eighteenth annual report, suffered financially from the prevailing depression last year; but on the other hand twenty fresh members have joined, and the sanitary tracts issued and the useful wall sheets have been widely circulated. As times improve doubtless the old subscribers will gladly come forward again, and the excellent course of lectures, for which fees are paid, will assuredly be as well attended as the free courses have been. At the eighteenth annual public meeting, held in the Assembly Hall at Melbourne, three "lecturettes" were introduced, and were much appreciated by a large audience. The subjects taken were "The Influence of Tea-Drinking on Health," by Dr. Bennie; "Dangers of Indoor Life," by Dr. Louis Henry; and "A Common Bad Habit," by Dr. Neild.

### SHORT ITEMS.

The examinations in elementary and professional instruction have been passed by 467 pupils from the male and female nursing staffs of the Paris hospitals, and 334 diplomas will be given.—The *Colonies and India* says that twenty Canadian girls training as nurses in hospitals at Buffalo are to be sent back to the Dominion under the United States Alien Labour Laws!—An excellent concert at Didsbury has resulted in a pleasant addition to the funds of the Sick Nursing Association, for whose benefit it was given by various friends.—Gifts of books and periodicals will be gratefully accepted by the Barry (S. Wales) Nursing Association.—The Newcastle Cathedral Nurses have received £53 from an anonymous friend, for the purpose of laying out the garden round their Convalescent Home at Hexham.—The Nursing Guild at Hartlepool has held its fourth annual meeting, at which it was announced by the Mayor that the prospects and balance of the association were much better than they have hitherto been.—Nurse Agnew, who was trained at the Crumpsall Infirmary, Manchester, has been appointed nurse for the sick poor of Ballycastle.—The Kettering Industrial Co-operative Society has given a concert in aid of the funds of the District Nursing Association.—A new association is about to be formed, under the title of the Beith Parish Nursing Association. It will be affiliated with the Queen's Jubilee Institute.

### On General Nursing.

By Rowland Humphreys, M.R.C.S., L.R.C.P.Lond.

### III.—SCARLET FEVER.

### Appearance of the Rash.

Scarlatina, or scarlet fever, derives its name from its most prominent symptom, the very bright colour which is one of the peculiarities of its rash. It is of a decidedly brighter tint than measles, and the spots run together from the first, and do not, as in the other complaint, remain separated. It is distinguished from "German measles" by the spotty rash with which the latter begins, and by the mildness of the premonitory fever; though the shortness of the prodromal period and the appearance of the fully-developed rash is similar in both complaints.

Scarlatina exercises, fortunately, a very wholesome terrifying influence on the public mind, and the result of this is that isolation is, in most cases, efficiently carried out without any unnecessary obstacles being thrown in the way.

The chief characteristics of the complaint are: (1) Its incubation and prodromal periods, both of which are remarkably short; (2) the suddenness of the invasion; (3) the sore throat and rash; (4) the completeness of the desquamation; (5) the tendency to renal complications.

### The Period of Incubation.

The first manifestation usually makes its appearance some twenty-four hours after catching the disease, but the period of quarantine for a person who has been exposed to the infection must be prolonged to eight days, as it occasionally makes its appearance so late as this.

### The Prodromal Period may be altogether absent, the rash being the first symptom. It is always short, so that the rash appears on the second or third day of the illness.

### The Invasion.

The first symptoms are well marked, usually headache, with vomiting, sore throat, foul tongue, a very rapid pulse, and an increased respiration and temperature. The pulse rate is increased out of all proportion to the rise in temperature or respiration. For instance, the temperature in one case was 99·2 Fahr., with a respiratory rate of 26, and a pulse of 140 instead of 130 or less, as we should expect from the respiration. In severe cases the temperature may be at or below the normal. The face is flushed and the neck sore, the glands at the angles of the jaws being swollen and tender. The skin may be hot and dry, or perspiring freely, and where there is much perspiration the skin becomes rough with miliaria, between whose little prominences the skin may have a faint flush. The throat is red and swollen internally, the soft palate being frequently speckled with red points, and the temperature soon runs up to 104 or 105 Fahr.

### The Rash.

At the end of a short period, varying from a few hours to three days, the rash makes its appearance, first on the upper part of the chest or side of the neck, spreading steadily upwards and downwards, till, in the course of the next forty-eight hours, it has invaded all parts of the body. It reaches its height in from one to three days, and then, fading off in the same order in which it appeared, leaves the legs between the fourth and sixth days. The rash at first takes the form of minute red points, much the same as are seen on the throat; these gradually extend, and, running together, form a bright uniformly-distributed blush, which disappears on stretching the skin between two fingers, to reappear directly the tension is taken off the skin. As the rash appears the skin becomes swollen, due to its hyperæmic condition, and as this persists for a short time after the disappearance of the rash, and is especially marked in the eyelids and extremities, it may be mistaken for that due to renal inflammation, and cause considerable alarm. It disappears very quickly if not due to the complication mentioned. The rash does not always come out in the manner just described; it may appear in patches only, and this seems especially to occur in cases which have relapsed and the rash has appeared for a second time after a distinct peeling has taken place; on the other hand, it may come out, and after spreading for a short time, may disappear; it is then said to have "gone in," an event which is generally followed by serious consequences. On the other hand, it may appear or be mixed up with a petechial or hæmorrhagic rash, which also portends a very severe attack, indicating as it does the severity of the primary poisoning. In this respect we may remark that the quality of the infection seems to depend on the person from whom the disease has been caught; its results on the surroundings and health of the individual taking it.

As the rash comes fully out, the temperature and general symptoms diminish, and, if all goes well, there is no further trouble, the disease, so far as this individual is concerned, being at an end.

### Desquamation.

Peeling commences as the rash goes, that is about the end of the first week. It may appear first on the scalp, or eyebrows, but usually, following the same course as the rash, it commences on the sides of the neck and, extending upwards and downwards, reaches the hands and feet about the end of the fifth week, and by the end of the sixth it is completed. The last parts to peel are the webs and neighbouring skin between the fingers and toes. It is mainly through the particles of the shed skin that the disease is spread, and it is therefore of the highest importance to know exactly when it finishes. From many observations which have been made it has been found that this varies very considerably, and depends on the way in which the peeling process has been assisted artificially, on individual differences, and on the severity of the attack. The more severe the illness has been the more complete the desquamation, the thicker the skin is, the longer it takes to separate, the more frequent and thorough the bathing the more rapidly the process is completed, and the same applies to the use of ointments. The average time occupied in the process is six weeks, but with frequent and systematic attention to the skin it may sometimes be reduced to three weeks. The longest time it is necessary to isolate the patient is eight weeks. The skin from the heels takes longer to separate than this, but it does not seem to act as a medium for propagating the disease. Occasionally after peeling the new skin continues to come off, and gives rise to much unnecessary anxiety. The throat may also be the source of a fresh attack in another individual, and so long as any discharge goes on from, say, an ulcerated ear, so long is the patient infectious. The finer the flakes in which the skin separates the more dangerous is it to others, and the harder is it to convince the friends that peeling is going on; but in all these cases the skin comes off, and it will become more noticeable when the hands peel. Throughout the whole of the complaint the skin should be well rubbed all over at least twice daily with some ointment or oil, such as that which goes by the name of "camphorated oil." Many medical men use an ointment with five per cent. of carbolic acid in it; this is, of course, excellent from an antiseptic point of view, but is apt to be absorbed and produce the symptoms of poisoning by that drug. These symptoms closely resemble those of uræmia, to which these cases are particularly liable, in darkening the urine, causing vomiting, &c.; and the author has, on the other hand, used the oil mentioned with complete success in many cases.

*(To be continued.)*

### Nursing in Africa.

### A NIGHT IN A SOUTH AFRICAN ASYLUM.

A summer night, such as I love. A still, warm air, that will scarcely stir a blade of grass. An intensely clear sky, crowded with unnumbered stars, or flooded with the silver light of the moon "walking in brightness," except for the incessant harsh metallic croaking of the frogs in distant pools and marshes, and the perpetual whir of millions of grass-hoppers and crickets, Nature seems to be all asleep. "On such a night as this"—give me night duty.

For there are many nights that are not such as this, especially in this windiest of windy places. Nights when every door bangs, and every window rattles, and every lamp blows out; when the rain drives on the iron roof of the verandah like a storm of small shot; when one's head grows weary and confused with the continual steady roar of the wind, rising now and again into a furious, shrieking gust.

And there are nights when the wind drops, and the clouds lie on the hills, and all night long one vast sheet of water descends from heaven, with the roar of a never-ceasing cataract. This is called a "beautiful rain."

There are frosty nights, too, in winter, when the outlines of the hills seem cut in black marble, and the stars are points of fire—when the whole landscape is white, reminding one of October mornings at home. But this is not one of these nights; it is a sweet, balmy, indolent summer night, when the earth rests from the blinding sunshine and quivering heat of the day.

It is already late, and the patients have been some three hours in their beds, so that by rights they should, most of them, be quietly asleep. But this is not likely to be the case, for as the nights are, so are not the patients. On cold, stormy nights they curl up under their blankets, and are as quiet as a pack of mice; but on these warm, still nights, and especially when the moon is bright, they are apt to be all awake and lively, sometimes the greater part of the night. This is more particularly the case with the natives who are accustomed, in their own homes, to dance and sing the whole night through on moonlight nights. On a Saturday night, if it is fine and warm, the festivities of the natives in the Kaffir village or "location," on the other side of the valley, can be very distinctly heard in the asylum; and this naturally excites the native patients, who "keep it up" in emulation. So that I am not in any way surprised or startled when I hear, in the further end of the building where the natives are sleeping, a noise like distant claps of thunder—first a heavy thud, followed by two lighter ones, then another heavy one, and so on. It is a native woman indulging in a Kaffir dance, and the noise of her bare feet on the wooden floor of her room, accompanied by tremendous hand-clapping and a loud monotonous Kaffir chant, is absolutely deafening when you are in the immediate neighbourhood. As it is quite sufficient to wake up nearly every patient in the building, I must pay her a visit and put a stop to her antics. For in this asylum, so different to an English one, where the floors and partitions are thin, and every part is full of doors and windows, which stand open all night in the summer, every sound can be heard throughout the whole place, which is, on the whole, rather a convenience for the night nurse.

The native patient in question is not often bad-tempered; she is only the very incarnation of mischief—mischief gone mad—and as soon as she hears the key turning in the lock she leaves off dancing and whisks into her blanket, and by the time I am in the room she is covered up, head and all, laughing heartily in enjoyment of the joke. I administer a scolding, of which she does not understand a word, and she replies volubly in Kaffir, of which I do not understand a word; and having arrived at this satisfactory result I leave her.

Every hour the night nurse makes the tour of the building, recording her visits on clocks stationed at certain intervals. It is a very simple affair, for each dormitory is the width of the whole building, so that they are all in one line, opening out of each other. First in order come the European dormitories, and after them the native dormitories, the last of which present a strange appearance to unaccustomed eyes, consisting of a very large room, perfectly bare. Here are between fifteen and twenty native patients, who are noisy, troublesome, destructive, and who, not being over-burdened with civilisation, have perhaps never slept on a bed in their lives. Here they sleep, as they would do in their own huts, each one lying on the floor rolled up in a blanket, so that the night nurse on entering sees nothing but a succession of mounds, like human ant heaps, with here a leg or an arm projecting from under the blanket, and there a black woolly head.

These native dormitories are provided with shutters, as the windows open directly on the verandah roof, and some of the patients have a propensity for breaking the windows and taking a midnight stroll, like cats, on the roof.

On leaving the dormitories, which are upstairs, I proceed downstairs, where two long corridors run nearly the length of the building, with single bed-rooms on the one side and a day-room at either end. The day-rooms being like the dormitories, the width of the building, have doors on either side opening on to the verandah, or stoep as it is here called; and it is my delight on a warm, still night to stand outside drinking in the beauty of the night, and watching the gradual changes that every hour brings in this nocturnal world. Sometimes there is an exquisite moonrise to watch, the pale yellow light as it spreads over the sky, gradually whitening to a pure silver, while one horn of the moon, blood-red at first, peers above the dark line of the hilltop. Sometimes there is incessant lightning playing about a dark mass of cloud on the horizon, reminiscence of a storm that has passed away or prophetic of one to come. And always there is the ceaseless, silent march of the constellations passing on their everlasting way, "without haste and without rest."

Never do I stand here at night, even when the sky is black and starless and the outlines of the hills are scarcely visible, without thinking of that wonderful line of Keats'—

"There is a budding morrow in midnight,"

and of all the meaning it conveys.

Between twelve and three is usually the quietest time. The noisy ones have tired themselves out, or a soothing draught has had the desired effect; and even the earliest of the "early birds" are not awake yet. Sometimes we are disturbed by unearthly sounds from the male asylum across the way; sounds which, on a still night, will seem so near that I am seized with terror lest a male patient should have escaped and be coming to pay me a nocturnal visit. Many a time, too, the unspeakable yellings of stray cats or dogs prowling outside have sent me flying through the building, under the impression that one of my patients was being killed at the very least.

There are one or two patients who, if wakeful, object to being disturbed by my periodical visits with the lantern. One of these asked me one night the pertinent question, "Nurse, how much do you get a month for disturbing the peace?"

But I must not linger for it is dawn already. Patients who sleep early are waking, and beginning to chatter or sing. Outside, before it is light, the little Christmas beetles, as we call them, begin their strange whizzing noise up in the trees, something like an electric bell that never stops ringing. Meanwhile the sky begins to put on its glorious garb of morning—colours too lovely and delicate for any words to describe, too fresh and wonderful, on each succeeding morning, for one ever to weary of the spectacle. "Custom cannot stale its infinite variety." I cannot stay to watch it as I would like to do, for the morning brings work, and various small tasks fill up the next two hours.

Half-past five! I know from my own past experience what blessings are invoked on my head as I knock at each door in turn and rouse the day nurses from their well-earned slumbers. Such consolation as I can, I next prepare for them, in the shape of "early coffee," which is an universal institution in the colony, where all are early risers; and lastly, I must tax my memory for the events of the night, and duly record the same.

Now, it is past six o'clock, and my last round is accomplished; the sun is already hot, the world, both without and within the walls, is up and stirring; the labours of the day have begun and those of the night are ended—I am "off duty."

A. H.

### The Training of Male Nurses.

REGRETS are constantly expressed at the absence of facilities for training male nurses in England. It may, therefore, be of interest to many readers of THE HOSPITAL to learn something of the scheme which has been long and successfully working at the Deacons' Institute, Druisberg, on the Rhine —Pastor Engelbert, the director, having kindly furnished us with full details of its organization.

In the forty years' development of the institution it has embraced not only the care of the sick but of the poor, of destitute children, and of prisoners. Young men have been definitely trained for each of these services, but more especially to take charge of the sick, for whom a large hospital—connected with the Deacons' Institution—has been established.

The period during which the probationers remain in the institution is variable, and is regulated by each individual's circumstances, or by the special needs of the hospital.

The young men receive instruction from the inspector and the head assistants; the latter, being candidates for ordination, are employed in supplementing the very elementary education already possessed by most of the probationers. They teach Church history, the Catechism, and explain the Scriptures, as well as the history and present organization of Home Missions.

From the candidates the director selects for the service of the sick those who show particular aptitude for such work, and they are transferred to the hospital, where, after a certain period of training, they are placed in responsible posts, and pledged to continue their studies and their own development.

Whilst they are in hospital they are under the control of the house governor (Haus Vater) and the medical staff, and receive their first instruction from the head nurses. Theoretical, as well as practical training is given during their residence in hospital, and they are expected cheerfully to undertake all duties required of them, including the cleaning of wards, floors, windows, and utensils.

A deacon and an assistant work together in district nursing, and accompany the doctors on their rounds, receiving instructions direct from them. As soon as they prove themselves to be reliable they assist at operations, and in other ways have the same training as that given to female nurses.

Theoretical teaching is given by the medical staff, and comprises the treatment, care, transport, and feeding of the sick; anatomy, clinical instruction, and occasional demonstrations in the dissecting-room.

Besides being employed in the hospital, men are engaged by the local authorities as district nurses. They also take posts in private hospitals. Chiefly those under the direction of deacons trained at the same institution. They also become private nurses, and those deacons who have accomplished several years of satisfactory work in the institutions are generally promoted to responsible independent posts, such as superintendents of training schools, house governors in hospitals, &c.

Applicants for training must be of the Evangelical persuasion, and be possessed of a Christian frame of mind, a living faith in their hearts, and an earnest desire to serve their needy brethren. They must not seek admission from any wish for worldly advancement or social promotion. They must be unmarried, unbetrothed, and, if widowers, they must be without encumbrances or claims on them. When accepted they must pledge themselves to remain unmarried for five years, and to give three months' notice before terminating their engagement with the society. Candidates are admitted between the ages of eighteen and forty, and they cannot select beforehand the special branch of work in which they will be employed. They have to present to the society, before admission, a written history of their previous lives, including a list of books recently read, and the names of pastors under whom they have worshipped, stating in both cases from which volume or preacher they have derived most edification. The young men have to produce certificates of birth, baptism and confirmation, a testimonial from last employer or teacher, and one from their pastor. They must also show that their application is made with the knowledge and permission of their parents, and give detailed and satisfactory proof of their own willingness to devote themselves entirely to the work which they aspire to share. When the deacons are employed as private nurses the institution receives two marks (rather less than two shillings) a day and travelling expenses—but the men themselves get no pay, any presents received by them have to be immediately handed over to the institution.

The duties of a deacon to the private patient include sweeping and otherwise keeping in order his room as well as the apartment which has to be allotted to the attendant's separate use (unless the exigencies of the case necessitate his sleeping in the sick man's room). He is required to be cheerful and obliging towards the rest of the household; but his duties are strictly confined to attendance upon the sick, although he has permission to go to the chemist, or fetch pastor or doctor in a case of emergency. When attendance on a patient is necessary by night as well as day, three or four hours' unbroken sleep must be secured to the deacon every afternoon, and he must have each third night undisturbed. One hour's exercise in the fresh air daily, and one attendance at a Sunday religious service, are also stipulated for.

If the deacon does not take his meals in the sick room he is always to have them alone. If any male nurse proves unacceptable to a patient the friends can ask for another to be substituted, but no deacon is allowed to remain more than three months with the same case.

### Notes and Queries.

### Queries.

(25) *Trained.*—Will you kindly inform me whether the matron of a Nurses' Home need be herself a trained nurse?—*Sister Josephine.*

(26) *Training Wanted.*—A respectable young woman wants information as to training.—*C. P.*

(27) *Our Examinations.*—I should be glad to know if THE HOSPITAL Examination Questions may be answered by other than trained nurses. —*Miss E. P.*

(28) *Cottage Hospitals.*—Is there any diet sheet published specially for use in cottage hospitals?—*Matron.*

### Answers.

(25) *Trained (Sister Josephine).*—It is better that she should be, otherwise she must be content to entirely confine herself to the housekeeping and general management, and have an experienced, fully trained, and certificated lady as superintendent of nurses and patients.

(26) *Training Wanted (C. P.)*—Get "How to Become a Nurse," by Honnor Morten. We think you are too young for a General Hospital.

(27) *Our Examinations (Miss E. P.)*—Certainly, the questions may be answered by any of our readers who like to do so.

(28) *Cottage Hospitals (Matron).*—Yes, on page 201 "Cottage Hospitals," by H. C. Burdett, second edition; cost per patient, &c., page 81. Published by Scientific Press, 428, Strand.

### Women's Work.

### TYPEWRITING.

By A. A.

The advice usually given by a cynical bachelor to a friend about to marry is, "Don't." Now, I am not a cynical bachelor, but an experienced "old maid," or rather, shall I say, "an unappropriated blessing?" And I say to my girl friends, to those at least who are about to learn typewriting in order to make it their profession, "Don't," unless—and on that word hangs everything—unless you mean to go in for it thoroughly and become absolutely efficient. You must get beyond the average, for sorrowfully I must confess that the average is bad, very bad. There is nothing like proficiency if you want to get on in the world. You have got to do one thing better than all others before you can claim that the world owes you a living. Therefore, aim to be first class. If that be your intention, take up typewriting by all means, for there is plenty of room for you; in fact, you are wanted badly. For the inefficient, incompetent stenographer and typist—and their name is legion—there is no room; they will assuredly be crowded out.

By typewriting, of course, I mean typewriting and shorthand, for the one is the natural complement of the other; they are indeed "twin arts." I would not advise anyone to be a typist only if she wishes to make a good living, for the average pay of a typist is from 15s. to 25s. a week; 30s. is the exception, and a rare one, by no means the rule.

"To be or not to be, that is the question," and having made up your mind on the point, the first question that confronts you is, "How and where shall I get my training?"

Now, of course, it must be first understood that a good general education is absolutely necessary, punctuation and spelling are of supreme importance, as also is punctuality. If you know German or French well, or German and French, so much the better, for your value as an amanuensis or secretary will, by the knowledge of those languages, be very much enhanced.

Now to come back to this question of training, I should say that a good typewriting office is unquestionably the best place, for many obvious reasons. There the work is systematic, regular, and practical; you see all kinds of work, and at the same time the routine of an office would get you at the outset of your career into good business habits. Women so often compare unfavourably with men in this respect, for, as I saw in an article some years ago, "A man grasps his business with both hands, if his hands are not strong enough he clamps it with his feet, and rather than let it go, he seizes it with his teeth. It is his life—himself."

Now that is what we must do; it is thoroughness we so often lack; if I did not recognise that fact I should indeed be a short-sighted champion of my sex. We must undertake our work with the intention of performing it, as if it were the one and only object in life.

There are many typewriting offices in London, and their numbers are greatly increasing year by year, and in almost all the biggest towns of England there is one, if not more. The terms for instruction in offices vary but little; in most cases they are the same, viz., £5 5s. for shorthand, and £2 2s. for typewriting—£7 7s. in all. This is surely very little when we come to consider what it means to us. It means that for £7 7s.—in some cases £6 6s. or even £5 5s.—we can procure the necessary equipment to enable us to be independent and earn our own living. It has always seemed to me so unjust that in numberless instances parents think nothing of the money they spend on their sons after they leave school, in order that they may be trained for whatever profession or business they are going to follow. But when it comes to the daughters it is quite different; they so often exclaim, "Dear me, and after I have spent so much money on your education; what more do you want?" in a most aggrieved tone.

Another reason why I advise an office is this: When a pupil is competent and ready to take a position she can almost always get one through the office in which she has been trained, as employers, instead of advertising for a clerk, secretary, or typist of any kind, find out the names and addresses of different offices and apply there to the principal, who is only too glad to place her pupils in good positions.

As regards the question of time, a girl of average ability and ordinary perseverance ought to learn both shorthand and typewriting in from six to nine months, that is, of course, if she works the usual office hours; but I would advise her, if it were possible, to stay twelve months in the office. It would not be time wasted, for she would be gaining a certain amount of all-round experience that would be of inestimable advantage to her in her after career.

The necessary speed varies so much, according to the work to be done, that I cannot speak definitely. For instance, a reporter would require a very much higher speed than a secretary, amanuensis or ordinary clerk, and also, whereas one man may dictate his correspondence at the rate of eighty words a minute, another might dictate at 120, so that this is a point which every individual would have to find out for herself; but, generally speaking, I would not advise any girl to take a position until she could write at least ninety words a minute.

Some of the mistakes that stenographers and typists make when taking down and transcribing their notes are very ludicrous. A gentleman whom I know was dictating a short time ago to his secretary a letter, in the course of which he said, in relation to a scheme which his correspondent had submitted to him, "Really, you are a wild Utopian;" this was taken down and transcribed as, "Really, you are a wild Ethiopian," which was very funny, to say the least of it. Of course, the girl had misunderstood what he had said, but at the same time her common sense ought to have told her that Ethiopian was not the right word. I have only related this little incident in order to show how easily mistakes are made, and now necessary it is to be careful in transcribing notes.

Finally, I say to you, be thorough, then you need have no fear as to results.

### Women's Volunteer Medical Staff Corps.

On Saturday last a meeting was held to promote the establishment of the above corps, Mrs. Heatherley being in the chair. Brigade-Surgeon Lieut.-Colonel Evatt very kindly gave an address explaining the duties of the Medical Staff Corps in war, dwelling on their arduous character. He did not speak as a supporter of the new movement, but in order that all who joined the proposed corps might thoroughly understand the nature and difficulties of their undertaking. When asked whether his experience led him to believe it possible for women to form an efficient corps, Lieut.-Colonel Evatt replied that he found the work demanded the very strongest men, but that it remained with women to prove whether they were capabie of it or not. Mrs. Heatherley and Dr. Alice Vickery spoke in warm approval, and supported the idea that the discipline and exercise entailed would be excellent for women. Several members of the Volunteer Medical Staff Corps attended the meeting, and while doubting whether women could bear the whole training their corps undergoes, expressed sympathy with the movement if carried out on practicable lines. Several members made kind offers of help. Names of those willing to join were then enrolled; the number of recruits is at present about twenty; but it has been decided to commence training at once, delay being caused only by the making of the necessary preliminary arrangements. The practical point at issue, Whether women's physical strength is sufficient at present to enable them to form an efficient Medical Staff Corps? can only be determined by practical experiment; but by the kindness of those who sympathise with the movement, a free field is afforded for the trial, and it rests with women themselves to prove what they can do.

### Nursing in the United States.

OUR NEW YORK LETTER.

THE managers of the Bellevue Training School issued their annual report early in the year, and it certainly was a most satisfactory one. They have now some four hundred graduates in various parts of the world, sixty of whom occupy positions as superintendents or head nurses in hospitals.

Miss Allen, the late Matron of the City of London Lying-in Hospital, was trained at Bellevue; and so was Miss Lett, Superintendent of St. Luke's Hospital, Chicago, who also died last year.

Lectures have been given to the graduates in the school by Dr. Robert Carlisle, on "Diseases and Symptoms"; Dr. Herman Biggs, on "Physiology and Fluids of the Body"; Dr. Jasper Garmony, on "Surgery and Emergencies"; Dr. Robert Wylie, on "Obstetrics and Electricity." Miss Agnes Brennan and Miss Carrie Brink (the Superintendent and her assistant) have held weekly classes, and there has been a course of lectures on "Sick Cookery."

The Edith Home, a charming resting-place, was again placed at the disposal of tired nurses during the summer months by the kind courtesy of Mr. Northcote, and many more applications for admission were received than could possibly be entertained.

The address to the graduating class was given by Professor M. Allen Starr, M.D., and was a peculiarly interesting one. Amongst other apposite remarks, he warned the nurses "that you must keep the knowledge you have gained well in hand by review and by application to constant use. Do not go out of this school, then, feeling—as college students often feel—that you know everything, that your training is complete; no one's training is ever complete. It is the observant man who gets something out of everyone and everything who succeeds; and you must continue your training just as long as you continue your work, training yourselves in a thousand ways, as no one else can train you." "Try to keep well rather than to get well," and "in taking care of your bodies do not neglect your minds" were amongst the valuable pieces of advice contained in Professor Starr's admirable address. The period of training at the Bellevue Hospital is two years, and the candidates preferred are those between twenty-five and thirty-five years of age. and holding a certificate of "sound health" from a physician before their admission. The superintendent has full power to decide as to their fitness for the work, and the propriety of retaining or dismissing them. At the conclusion of the two years' training there is a final examination, after which diplomas are granted, and the nurses are at liberty to choose whether they will in future take posts in hospitals, in district work amongst the poor, or in private families.

### Novelties for Nurses.

UNSHRINKABLE FLANNELS.

WE have received some wonderful flannels from Messrs. Barker and Moody. For softness and substance they are really incomparable, and being absolutely unshrinkable will stand any amount of wear. It is unfortunate that no prices are quoted, therefore it is impossible to judge of their relative cost, but for quality certainly nothing could be better. Particular mention should be made of the "Specialité" flannels, which are in pretty colours, and will make charming dresses. The white flannels are excellent, too, in many different qualities, while some of the checks and stripes are extremely pretty as well as serviceable. These flannels can only be obtained through outfitters and drapers, not direct from the manufacturers, who, however, will be pleased to supply the name of the nearest draper who keeps them on any application to Perseverance Mills, Leeds.

### For Reading to the Sick.

DEPENDENCE ON GOD.

**Motto.**

That which I see not teach Thou me.
—*Job* xxxiv. 32.

**Verses.**

There is a crook in every lot,
And a need for earnest prayer;
But a lowly heart that leans on Thee
Is happy everywhere. —*J. P. W.*

I make me cords to hold from wrong,
And bind my will by purpose strong;
But my resolves, as cords of tow
Before the strength of passion, go
Like hempen bonds which flames o'er-run,
Or icy streams before the sun . . . .
Lord! Who hast ta'en us by Thy Hand,
'Tis only by Thy strength we stand.
—*L. Williams.*

**Reading.**

We must learn more and more to depend upon God if we wish to serve Him faithfully. Many persons spend years in trying to make themselves good without God, and, though He accepts every honest effort, yet they fail miserably. We make more progress in one year, when we have learned to depend upon God, than in a whole lifetime of struggling by ourselves. It must be so, for Jesus Christ has said, "Without Me ye can do nothing." "In Him we live and move, and have our being." This dependence on God is taught in the Bible everywhere. Take, for example, such an illustration as the little child. We are told to become as little children, and if we do not we cannot be Christ's disciples. This seems to be a strange thing to say to grown-up men and women, to kings and queens, to wise and learned counsellors, and great warriors. It seems strange to say to them, "Except ye be converted, and become as little children, ye cannot enter into the kingdom of heaven." And what is there in little children that made our Lord point them out as illustrating the very thought which He wanted to teach humanity? Look at them in their helpless infancy. Do they not seem to be waiting till God gives the power to walk, and to speak, and to think? What a picture for our guidance! Every hour, every moment, depending upon God, not rushing into a thing, but waiting till God gives the word, waiting that God may guide us, and then going forth calmly, and powerfully because calmly, to act, depending upon the help of God. . . . The whole discipline of life also teaches us this lesson of dependence. How little we know what a day may bring forth, what may happen to us in our body, or what may happen to us at our work, how it may be altered, how we may be taken from it, or it may be taken from us. Does not this make us depend necessarily upon a Higher Being? This again is the meaning of many of our failures. Go over your life, the good that you have tried to do, and the people that you have had to mix with, how the health broke down perhaps, or how the effort all came to nothing, and the resolutions all seemed to end in hopeless defeat, how you have broken down in body, soul and spirit, and at last only found rest by saying, "Yet not I, but the grace of God that is in me." . . . . What is God doing through it all but teaching us dependence upon Him, as to what He may see fit at any given moment to bestow upon us? The whole life of man, from the child in its mother's arms, to the old man tottering to his grave, is designed to teach us this lesson, dependence upon God. Sin has come in here, and the object of this life's discipline is to train us day by day to "Rest in the Lord, and to wait patiently for Him."—BISHOP OF TRURO, "*Communion of Saints.*"

### Australian News.

### OUR SYDNEY LETTER.

The new buildings of the Sydney Hospital are rapidly approaching completion, and will be ready for occupation before the end of the year. They are situated in the city in Macquarie Street. We have no properly-constituted maternity hospital in Sydney, a fact which is much to be deplored; but for many years lying-in wards have formed part of the Benevolent Asylum, which stands near the centre of the city in one of the main thoroughfares. Towards the latter end of 1893 puerperal fever made its appearance in these lying-in wards, which are situated in an old building, and although the committee did their best to thoroughly purify the place they did not succeed in entirely banishing the disease. Differences arose between the honorary and paid medical staff, with the result that the former resigned in a body. This led to a little newspaper warfare, with the result that new quarters have been provided, the present lying-in wards being at Redfern, a suburb within convenient reach of the city. Four fresh honorary medical officers have been appointed. Miss Grace Robinson, M.B.Ch.M., one of the first two lady graduates of the Sydney University Medical School, has been appointed resident medical officer of the new department. It is still worked as a branch of the Benevolent Asylum, and we hope that the change will be productive of much good; at all events, it is a step in the right direction, and one by which better instruction will be secured to students and nurses.

Last December Sir Alfred Roberts had an awkward fall whilst attempting to enter a steam car, and sustained a fracture of the neck of the left femur. Despite his somewhat advanced age and the very trying weather he has had to contend with, he is progressing so favourably that we have every hope of seeing him about again soon. As he is honorary secretary to both the Prince Alfred Hospital and the Carrington Convalescent Hospital at Camden, his absence from both committees has been greatly deplored. Sir Alfred's interest in hospital work is well known, and he is acknowledged to be the greatest authority on hospital construction and management in Australasia.

Miss McGahey who was trained, and afterwards held the position of sister at the London Hospital, still ably fills the responsible position of matron and superintendent of nurses at the Prince Alfred Hospital.

Owing to the wave of depression which passed over the colonies during the past year, the subscriptions did not come in satisfactorily, and the committee thought fit in the middle of the year to reduce all salaries]10 per cent. This was not received very pleasantly, since the different officers are not paid too well at any time. After the balance-sheet is made out, however, we think that the financial condition will compare favourably with last year.

At the Sydney Hospital they had to meet the same difficulty, with a much larger debt staring them in the face, but the committee only made a few minor reductions, leaving the majority of the salaries of the hard-worked officials untouched.

Mr. F. J. T. Sawkins, M.B.Ch.M., who had been senior resident medical officer for a period of one year, and prior to that house surgeon and house physician respectively, and is a graduate of the Sydney University, now holds the post of medical superintendent at Prince Alfred Hospital.

Dr. Cecil Purser, so well known for his able administration, resigned in October of last year to start in private practice in Lewisham, New South Wales, a suburb within a few minutes' railway journey of Sydney. When Dr. Cecil Purser left the hospital to which he had rendered such excellent service for four years, he was presented by the resident staff with a full-size roll-top walnut desk and a bookcase, both of which bore on silver plates the inscription, "Presented to Dr. Cecil Purser, by members of the staff of the Prince Alfred Hospital, on the occasion of his retirement from the position of medical superintendent."

### Where to Go.

Royal Institute of Painters in Water Colours.—The annual exhibition of this society opened on Friday last to its friends and members, and upon Saturday to the public. The usual conventional school of English water colours is represented by its well-known exponents, the President, Mr. Bundry, Mr. Gordon Browne, Mr. Catermole, &c. In the western gallery we noticed an excellent piece of tone-painting by Mr. G. Straton Ferrier, a sunset effect by Mr. Severn, and Mr. Nisbet has a well-treated but monotonous piece of moorland. One of the best works this year is Mr. J. Austen Brown's "Weed Burning" (105), which though distinctly recalling J. Francois Millet is still original, and invested with a charm which no mere copyist of a school could attain to. Next to this picture we find one of the six charming landscapes which Mr. Aumonier sends, quite up to his usual form, and each one a study in itself. Mr. Hayes and Mr. Ingram give us two clever bits of sea painting under different atmospheric conditions. In the central gallery, Mrs. Cotman's "Steaming into Lincoln," a scene on a railway embankment, is good; Mr. Tuke's sketch of a Cornish fisherman is worthy of notice; Madame Ronner is represented by two of her inimitable studies of cats; and Mr. Muirhead by a quiet bit of colouring in his "Grey Day on the Thames." We would especially recommend to notice Mr. Hansen's exceedingly clever sketches (291 and 376), full of life, light, and, though small in actual size, they are broad and large in composition. Mr. Wetherbee has a good study of "Gleaners," though it is rather wanting in drawing. In the east gallery is a fresh and crisp study on the "Quai des Fleurs," by Mrs. Bayes, who also sends a tennis match of French Priests (624), which forms quite an oasis in the desert of conventionality. Not only is the picture strong in treatment, but the framing of it is artistic, and being so is presumably "home made." There are a few miniatures, but none of any great merit.

St. Jude's Schools, Commercial Street, Whitechapel.—Free exhibition of pictures opens on March 20th. Professor Herkomer will deliver the inaugural address.

Medico-Psychological Association of Great Britain and Ireland.—The next examination for this nursing certificate will be held on Monday, May 7th, 1894. Intending candidates should apply to the registrar of the association (Dr. Spence, Burntwood Asylum, Lichfield) for schedules, which, when filled up, should be returned to Dr. Spence not later than Monday, April 9th.

### Appointments.

District Schools Infirmary, Sutton, Surrey.—Miss Louise Thornton has been appointed Matron to this infirmary. She was trained at Bristol General Hospital, and afterwards held the post of Sister and Night Superintendent at Swansea Hospital, and Matron of Swansea Borough Hospital. Miss Thornton has good testimonials, and we wish her every success

County Infirmary, Carlow.—Miss G. E. Spencer has been appointed Lady Superintendent of this infirmary. She was trained at Crumpsall Infirmary, Manchester, and was afterwards for two years Sister at the Nursing Institution and Memorial Hospital, Mildmay, London. Miss Spencer was Night Superintendent at the National Hospital, Bloomsbury Square, and Assistant Matron at the Royal South Hants Infirmary, Southampton. We wish her every success in the new work she has undertaken.

Queen Charlotte's Hospital.—Miss McCord has been appointed Matron of this hospital. She was trained at the Crumpsall Infirmary, Manchester, where she qualified for the diploma of the London Obstetrical Society. She was Night Superintendent at Crumpsall Infirmary, and also at the City of Dublin Hospital, and for the last seven years has been Lady Superintendent at the Carlow General Infirmary, where she has done excellent work. Her varied experiences specially qualify her for the important post, to which she takes the good wishes of many friends.

☞ For Nurses' Looking Glass, Everybody's Opinion, and Book World for Women and Nurses, see page ccxxxix. et seq.

### The Muses' Looking Glass.

### "A DAISY UNDER ITS WING."

"Nursie, may I be quite close to the window, this side, so that I can see my little garden?"

"Yes, dear, of course," and the nurse pushed the small bed in front of the window and gently lifted the child higher so that he could see out. He was only seven years old, and he was very ill. The large blue eyes were unnaturally bright, and there were crimson patches on either cheek. A June sun streamed into the room; it tenderly kissed the golden curls which lay about the fair white brow.

"Nursie," he said again, "I do want to go out so badly; my garden wants digging up, and there are lots of daisies on the lawn to-day; yesterday you know there were only five. Do you think I might go out?" he asked wistfully. "And will you help me make a daisy chain?"

"Not to-day, dear; when it gets a little warmer you shall."

"But you say that always and I never go, and if you let me go I won't mind having that big shawl round my neck." The nurse bent down and kissed the eager little face, and said gently, "Not to-day, dear."

For a few minutes he struggled to repress the tears which wanted to flow, then he had a bright idea—

"Well, will you go and pick me some daisies, and I'll make a bunch for Mamma. Won't she be glad, because she told me she was going to some races to-day; and you know, Nurse, ladies always go grand to races. Don't you think she will like to wear a bunch of dear little daises?"

"Yes, I am sure she would. I'll go and pick them, and you can watch me from the window; and I'll see if anything fresh is coming up in your garden."

She left the room, and the boy looked out of the window and waved his hand to her, and smiled to see her gathering the small white flowers. He received them joyfully, and she propped him up with pillows and gave him some cotton. He was very happy. His fingers were childishly awkward, and the bunch took a long time to make. It was a very uneven bunch, but he held it up proudly, and said, "Look, Nursie; won't Mamma be pleased? Do you think she is coming to see me soon?" He had hardly finished speaking when the doors opened and a beautiful woman came in; she was exquisitely dressed, all in mauve, and a fragrance of lilac seemed to cling about her.

"Well, Harold, how are you? I think you are better." She bent and kissed her son, who flung his childish arms warmly round her neck. "My dear child, mind my hair," and she raised a jewelled hand to pin a curl which he had displaced. "Don't you think he is better?" she said, turning to the nurse.

"I am afraid not," she replied in an undertone. "He coughed fearfully nearly all night."

"Well, take great care of him, and if he—"

"Mamma!" in a shrill tone from the bed. "Look!" Triumphantly from under the sheet where he had hidden it, came the bunch of daisies. "They are for you!" He watched her eagerly to see how she would take such a delightful surprise.

"Thank you, dear child, they are very nice." Then she laid them down and went on speaking to the nurse.

"Mamma, you are going to wear them?" in a troubled voice.

"My dear Harold, I can't. Ladies don't wear daisies."

The tears rushed to his eyes. "And I made them into a bunch for you, and they *are* long stalks. Oh, Mamma, wear my daisies."

"I can't, really. You must not be a baby," for the tears were now flowing steadily down his cheeks. "It is time I was off. Good-bye, dear; I'll bring you home a new toy this evening."

She left the room. He held the rejected daisies tight in his little hand. Nurse kissed away his tears, and tried to make him forget his trouble. Then he had a bad fit of coughing; it exhausted him very much. For a long time he lay very still, with his eyes fixed on his canary, which hung just above his head. The bird sang very blithely to-day.

Then he spoke very quietly. "I should like my dickie to go to heaven with me; I have often seen little birds flying to heaven. May he go, please, Nurse? Because, you know, if I went without him I think he would miss me very much."

"Yes, dear, you shall take him with you; but you are not going yet."

"But I want to go soon; this pain is very bad, Nursie." And he placed a small transparent hand on his chest. "You told me I shouldn't have a pain when I got there, didn't you? And you said there'd be a big garden and I shall be able to pick daisies and buttercups all day; and without a shawl," he added, smilingly. "I'll pick you a big bunch and drop it down. I don't think my Mamma is fond of flowers," he said sadly.

Again he watched the bird hopping and bobbing on its perch.

"S'posing we let dickie go now? I shall know him directly I get to the sky."

"Oh, no, darling; you would not like to be without him to-morrow."

"Oh, yes, I should. He does want to go so, he whispered it to me, and you know Mamma says little birds whisper things to her sometimes. You get down the cage, Nurse; I'll take him out, and you shall open the window, and we'll watch him go to heaven."

He clapped his little hands with delight, and the nurse couldn't refuse. She unhooked the gilded cage, and he very gently took out the soft yellow thing. Then nurse opened the window, for it was quite warm.

"I'll put a daisy under his wing," said the boy joyfully. "Then I am sure to know him. Oh, but I forgot dickie flies with his wings, and he would lose it. Do you think my wings will be yellow like his?"

"No, I think they'll be white."

Tenderly the child held the bird. Lovingly he kissed its tiny head.

"Good-bye, birdie, good-bye; I shall see you soon;" but tears stood in his eyes at the prospect of even a short separation. Then he kissed it again, and gently stroked its wings. The bird did not struggle. He held it in the sun and opened his hand. For a minute the canary did not move. Suddenly it seemed to realise it was free, and with a joyous "tweet-tweet" it rose in the summer air and soared towards the deep blue sky. The sun danced on its golden plumage. The child watched it yearningly till it was out of sight, then he fell back exhausted, and closed his eyes—a smile on his pretty wan face. . . . He slept. The nurse drew down the blind to keep the sun off his face. But the blind didn't keep it off, and the pain in his little chest was gone. . . . He is picking daisies and buttercups now.

### Minor Appointments.

Keynsham Workhouse.—Miss Rose Cridland has been made nurse at this workhouse; she has not been specially trained, but has had experience as nurse at Chilton Union Workhouse.

West Ham Hospital, Stratford, London, E.—Miss Edith Davis, trained at the West Ham Hospital, has been appointed Sister of the Women's and Children's Wards.

### Everybody's Opinion.

[Correspondence on all subjects is invited, but we cannot in any way be responsible for the opinions expressed by our correspondents. No communications can be entertained if the name and address of the correspondent is not given, or unless one side of the paper only be written on.]

### NURSING SMALL-POX.

"A Bond Street Nurse" writes: I should like to call the attention of those of your readers engaged in nursing small-pox to the use of eucalyptus vaseline. My sister and myself have just nursed through a long and severe epidemic in the "black country," and we found that painting the patients' faces and hands, and in the worst cases the whole of the body twice daily had the effect of entirely stopping the irritation and preventing the smell so characteristic of small-pox. The patients made a much quicker recovery than is usual in such bad cases. The proportion of eucalyptus used was 25 per cent.

### HALF A CROWN.

"A District Nurse" writes: Having in the course of my duties to attend a woman just recovering from her confinement, and deplorably poor, a kind friend devoted to her service the modest sum of half a crown, which I spent as follows to the greatest satisfaction of my patient. I may add that I made the beef-tea myself, but the husband undertook the rest of the cooking. The articles purchased were—Hock of beef, 4d.; fish, 1d., on Monday. Fresh haddock, 2d.; egg, 1½d.; oranges, 1d., on Tuesday. Chop, 4d.; egg, 1½d.; cornflour, 1d.; Liebig, 4½d., on Wednesday. Hock of beef, 4d.; egg, 1½d.; fish, 2d., on Thursday. Plaice, 1d.; egg, 1d., on Friday. From these the following diet list was carried out:—

| | Lunch. | Dinner. | Tea. | Supper. |
|---|---|---|---|---|
| Monday ... | — | — | — | Fish. |
| Tuesday ... | Beef-tea. | Fish. | Egg. | Beef-tea. |
| Wednesday ... | — | Chop. | Egg. | Cornflour. |
| Thursday ... | Beef-tea. | Fish. | Egg. | Beef-tea. |
| Friday ... | Liebig. | Fish. | Egg. | Liebig. |
| Saturday ... | Liebig and cornflour over. | | | |

### ANNUAL LEAVE.

"A Nurse" writes: To benefit nurses who wish for a pleasant holiday, I send a short sketch of a week spent in Jersey (the largest of the Channel Islands) by myself and a friend. We were fortunate enough to secure comfortable rooms in a quiet part of St. Helier before starting on our journey. We left Southampton just after midnight, and had a smooth passage, arriving at Guernsey about half-past seven, where we stopped to unload innumerable fruit baskets. It was amusing to see the quick way in which they were handled by the men and boys, and also the clever manipulation of larger packages landed by the steam crane. While waiting we saw the Weymouth boat arrive with her passengers and cargo. Many people prefer crossing by the Weymouth route, on account of its being a shorter trip, but the difference is very trifling, only thirty miles less. On arriving at Jersey we had to carry our own luggage to a cab-stand, fares being about the same as in England. The streets of St. Helier are exceedingly narrow, with the exception of one or two near the market, which is itself well worth a visit. We soon reached the end of the town, and stretching far away to our right lay the beautiful bay of St. Aubins, in which Elizabeth Castle stands. It is an old fort, approachable on foot at low tide. We did not venture to pay it a visit during our first walk, preferring to wend our way along the pleasant esplanade, till we arrived at the bathing place near Cheapside Station. From thence we walked past the parade, theatre, gaol, and hospital, and so into the Rouge Bouillion, one of the best parts of St. Helier. Eventually we retired to rest with pleasant anticipations of the morrow. Next day we explored the eastern side of Fort Regent, which is built upon the rocks, and well garrisoned. Arriving on the sands we found ourselves in the pretty bay of Samarex, and after a pleasant walk, stopping now and then to pick up shells which took our fancy, we arrived at St. Clement's Bay, where we sat down to rest on a rock till the tide warned us to move on. Retracing our steps round Samarex Bay, past George Town into St. Helier, we were conveyed by 'bus to our rooms. The next day we took train from Cheapside to St. Aubins. From there we walked to Portelet Bay, climbing an immense hill on one side, then descending again to the sea. In this bay is an exceedingly pretty group of rocks, reaching out some distance into the sea, on the top of which is Janvrin's Tomb. It takes its name from a man who died of the plague and was buried there. We selected a cosy nook on the rocks and took our lunch, which we had brought with us. The tide again compelled us to move on, so we had a good scramble over the rocks looking for anemones in the little pools filled with sea-water, but the tide was relentless, and we had to beat a hasty retreat. We walked back to St. Aubins, and took the train to Corbiere, where the beautiful Corbiere Rocks are, on which the lighthouse stands to warn ships of the dangerous coast they approach. The wind was blowing very hard, and dashed the waves against the rocks with relentless fury, causing the spray to fly in all directions, and to smother the unwary who ventured too near. It was a lovely sight, and one we shall not soon forget. The next day we travelled along the eastern side of the island, by train, to Gorey, where is the beautiful old ruin of Mount Orgueil Castle. There is a most extensive sea view from the top of the castle, and on a clear day may be seen the white shores of Normandy. On our return journey we stopped at La Roque, where the pier is worth a visit, being cut through the beautiful red granite in which the island abounds. The remaining days of our stay we spent in visiting the various places of interest on the opposite side of the island by coaching tours. I must not forget to mention the wonderful Jersey cabbages, which grow to the height of six feet, and are converted into very nice walking sticks. They are spoken of as the Jersey coat of arms. It would take too long to enumerate the many places of beauty which we saw during our stay in the island. I can only recommend all who read this to go and prove for them selves if they do not enjoy it as much as we did.

### ANIMAL OR VEGETABLE?

"An Experienced Nurse" writes: I am very much puzzled by some of the expressions in a little book on hygiene which I have been studying lately, and in which germs are spoken of as "these beings," and again "these and all other germs are vegetable," and in another place the writer says that "beef tea . . . after being exposed to germs, swarmed with animal life." I want to know how a vegetable can produce animal life. Can this be answered in The Hospital?

[In speaking of bacteria as animals the writer of the book in question is simply guilty of making use of a slip-shod expression. Bacteria have for so many years been regarded as belonging to the animal kingdom, that it is somewhat difficult at the present day to disillusionise oneself of this belief. And this is the more easy to understand when one looks at living motile bacteria, under the microscope it seems almost impossible to regard them as anything else than living animals. Even the German bacteriologist, who is accurate if nothing else, habitually in conversation speaks of bacteria as "thiere" (animals), although he well knows them to belong to the vegetable kingdom.—Ed. T. H.]

### Wants and Workers.

A District Nurse tenders sincere thanks for old linen and under-clothing received for the poor woman with cancer. She will be very glad of a further supply. Mrs. M., 82, Victoria Street, Ashton-under-Lyne.

### The Book World for Women and Nurses.

[We invite Correspondence, Criticism, Enquiries, and Notes on Books likely to interest Women and Nurses. Address, Editor, The Hospital (Nurses' Book World), 428, Strand, W.C.]

Marie Charlotte Anne de Corday: A Centenary Monograph. By Mary Jaeffreson. (London: Digby Long and Co. 1893.)

"A hundred years after," says the authoress of this volume, "does not seem a very commanding position for reviewing anything, but to me it appears a very suitable distance from which to scan one of the beautiful meteor lights of that lurid period called 'The French Revolution.'" And Miss Jaeffreson is right when she considers that the story of Charlotte de Corday will bear a retelling in 1893. We have been so accustomed to think of this second Judith purely from the standpoint of publicity and political *entourage*, that it is a fresh interest to read of her in the way this little volume sees fit to do, and to understand the private secluded life of her girlhood, before Charlotte Corday took upon herself the mission which has immortalized her name. Miss Jaeffreson sets her heroine before us in all the strange incongruities which characterized her nature. "She was a child of her times," as her biographer puts it in a few expressive words, "in its backward movement, in the strange jumble of pagan and Christian models that moulded her, her girlhood's devotion to Plutarch's illustrious men, first obscuring and then altogether eclipsing the Christian traditions of earlier years" . . . . yet, later, in the dread tribunal, when she stood convicted of the crime she had perpetrated, "answering her judges with a candour that made them declare her true to the motto of her family, *Corde et ore*." The stain of blood-guiltiness cannot, indeed, be effaced from the record of Charlotte de Corday, but her latest biographer shows her to us, with much historic and narrative power, in all the glory of one who would free her people even at the cost of her own life.

The Story of a Stuggle.—A Romance of The Grampians. By Elizabeth Gilkison. (London: A. and C. Black.)

Miss Gilkison should not attempt the making of stories. The "purpose" in "The Story of a Struggle" is so strong that the plot is nowhere, and the characters are strained and unnatural, while the agony is unreasonably prolonged through three hundred and forty-three pages. James Stewart is a disagreeable Highlander, who deserts and thereby kills his early love, a sweet and bonnie lassie, who is sacrificed to the determination of James Stewart and his mother that the marriage of the former shall be a stepping stone upon which he may improve his future position as a "minister." The girl he finally marries is nearly driven desperate by his coldness and neglect, but after an attempt on her part to run away, and a hardly repressed inclination to murder on his, this unfortunate couple are mutually brought to see the error of their ways and live happy ever after. The authoress has evidently been animated by a real and laudable desire to benefit her readers by an attempted analysis of certain spiritual and mental conditions, but the best parts of the book are the descriptions of the beautiful Highland scenery, for which Miss Gilkison has evidently a true love and appreciation.

### MAGAZINES OF THE MONTH.

The Humanitarian for this month keeps up its standard of being up to date in the social problems of the hour. The treatment and training of "abnormal children" is in the interesting manner of Sir Douglas Galton. The Rev. Arthur Robins, who is well-known as a vehement advocate of the wretchedly housed poor, does not hesitate in his expressions of opinion in "Our Home Made Heathen." The article is very "strong" in parts, but his assertions bear the impress of truth and conviction, and will make many stand aside and think. A bright little article on 'Woman in Clubland" will be read with interest. The authoress takes a more hopeful view of the fitness of her sex to mix in a genial manner with her fellow woman in the free and easy manner of Clubland. If she can be educated to this, Clubland will have been beneficial in one respect at least.

The Westminster Review for March has managed to collect articles possessing more originality of thought and treatment than falls to the lot of the monthly reviews, however excellent they may be. Mr. W. R. Sullivan's "New Eirenikon" is one of these. He boldly stands out for the sweeping away of diverging creeds, and demands a religion wide enough to satisfy the demands of all nations and individuals of the earth. Mr. Sullivan infers that Christ's teaching in its full power and purity only exercised sway up to a certain period in history. It is not a new scheme that Mr. Sullivan suggests for adoption. We are to return to those times when the Master was teaching in person. In the light of an improved civilisation and of wider intellectual growth, we are to pick up the threads long since tangled by our ancestors, and follow them out in the straight and wide road of the religion in the paths of which they were intended to lead men. Yet from Mr. Sullivan's article it would seem that he would take us straight to Nazareth to reveal to us a God as much of his own creation as any god set up by the imagination of so-called heathens. The God he would have us find in Christ is Reason. We are to discard everything that cannot be explained by its light. Dr. Martineau seems to have been the leader most in accord with Mr. Sullivan's views. We doubt if he was ever as hopeful of the establishment of so definite and wide-spreading a standard of truth, however. Where and how this infallible standard of Reason and Truth are to be found, we are not told. If the working man and the most ignorant of our population cannot follow the simple lines of Christ's teaching, as expounded in our churches, how much less will they accept the "higher reasoning" in which it appears inevitable that each man will form his own standard and conclusions. And the gathering together of "men dispersed amidst strife and contradiction," as Mr. Sullivan puts it, will be as far off attainment as ever.

